# Supplementary material for: Assessment of different head tilt angles in volumetric modulated arc therapy for hippocampus-avoidance whole-brain radiotherapy
Source: Front Oncol. 2024 Jun 27;14:1415471. doi: 10.3389/fonc.2024.1415471 (PMC11236617; doi:10.3389/fonc.2024.1415471)
Supplement: Supplementary file 1 [file Table_1.docx]

Supplementary Material

Assessment of different head tilt angles in volumetric modulated arc therapy for hippocampus-avoidance whole-brain radiotherapy

Cuiyun Yuan^1†^, Sisi Xu^1†^, Yang Li^1^, Enzhuo Quan^1^, Dongjie Chen^1^, Jun Liang^1*^, Chenbin Liu^1*^

^†^These authors contributed equally to this work and share first authorship

*** Correspondence:**Chenbin Liu, [liu.chenbin@163.com](mailto:liu.chenbin@163.com)
Jun Liang, [liang23400@163.com](mailto:liang23400@163.com)

**Supplementary Table 1** Summary of the dosimetric outcomes for PTV

|  | **Group 1** | **Group 2** | **Group 3** | **Group 4** | **Group 5** |
| --- | --- | --- | --- | --- | --- |
| **PTV D_mean_ (Gy)** | 31.99±0.29 | 31.74±0.19 | 31.66±0.24 | 31.73±0.35 | 31.49±0.29 |
|  | p=0.016 | p=0.055 | p=0.109 | p=0.055 |  |
| **PTV CI** | 1.7±0.49 | 1.73±0.5 | 1.59±0.46 | 1.49±0.28 | 1.76±0.42 |
|  | p=0.383 | p=0.742 | p=0.313 | p=0.109 |  |
| **PTV HI** | 1.24±0.03 | 1.23±0.02 | 1.22±0.04 | 1.23±0.03 | 1.2±0.03 |
|  | p=0.016 | p=0.041 | p=0.071 | p=0.039 |  |
| **PTV D_2cc_ (Gy)** | 35.78±0.7 | 35.15±0.48 | 35.15±0.59 | 35.04±0.67 | 34.87±0.5 |
|  | p=0.016 | p=0.25 | p=0.148 | p=0.641 |  |
| **PTV D_98%_ (Gy)** | 20.15±0.51 | 19.89±0.29 | 20.23±0.98 | 19.7±0.59 | 19.91±1.1 |
|  | p=0.742 | p=0.945 | p=0.843 | p=0.25 |  |

**Supplementary Table 2** Summary of the dosimetric outcomes for organs at risk

|  | **Group 1** | **Group 2** | **Group 3** | **Group 4** | **Group 5** |
| --- | --- | --- | --- | --- | --- |
| **Cochea D_max_ (Gy)** | 32.23±0.49 | 32.10±0.70 | 31.38±0.86 | 32.01±1.12 | 31.40±0.93 |
|  | p=0.055 | p=0.109 | p=1 | p=0.313 |  |
| **Cochea D_mean_ (Gy)** | 30.83±0.50 | 30.69±0.65 | 29.73±1.29 | 30.37±1.19 | 29.83±1.04 |
|  | p=0.016 | p=0.109 | p=0.945 | p=0.25 |  |
| **OpticalNerve D_max_ (Gy)** | 34.10±1.17 | 32.60±1.25 | 31.86±0.52 | 31.49±1.66 | 30.17±2.94 |
|  | p=0.008 | p=0.039 | p=0.109 | p=0.313 |  |
| **OpticalNerve D_mean_ (Gy)** | 26.75±1.94 | 23.99±1.99 | 23.55±2.73 | 23.70±3.66 | 22.98±7.22 |
|  | p=0.547 | p=0.844 | p=1 | p=0.742 |  |
| **OpticChiasm D_max_ (Gy)** | 34.23±1.12 | 33.22±0.24 | 33.10±0.50 | 34.11±0.69 | 33.39±0.85 |
|  | p=0.109 | p=0.383 | p=0.461 | p=0.195 |  |
| **OpticChiasm D_mean_ (Gy)** | 32.27±0.59 | 31.31±0.83 | 31.35±1.03 | 31.44±2.39 | 30.91±3.41 |
|  | p=0.313 | p=0.195 | p=0.547 | p=0.148 |  |
| **Brainstem D_max_ (Gy)** | 35.36±0.82 | 35.65±1.14 | 35.02±1.07 | 35.45±1.03 | 35.33±1.01 |
|  | p=0.742 | p=0.742 | p=0.313 | p=0.945 |  |
| **Brainstem D_mean_ (Gy)** | 28.87±0.67 | 29.84±0.65 | 29.19±2.04 | 27.57±4.38 | 28.60±1.78 |
|  | p=0.844 | p=0.195 | p=0.461 | p=0.742 |  |
| **Pituitary D_max_ (Gy)** | 34.23±1.69 | 33.12±0.72 | 32.87±0.61 | 32.54±1.31 | 32.14±2.52 |
|  | p=0.039 | p=0.945 | p=0.844 | p=0.742 |  |
| **Pituitary D_mean_ (Gy)** | 31.46±2.41 | 31.40±1.18 | 31.99±0.67 | 30.73±2.28 | 30.81±3.48 |
|  | p=0.844 | p=0.25 | p=0.945 | p=0.641 |  |
| **Hippocampi D_max_ (Gy)** | 11.66±0.59 | 11.40±0.65 | 11.02±0.16 | 11.07±0.19 | 10.73±0.36 |
|  | p=0.008 | p=0.016 | p=0.148 | p=0.039 |  |
| **Hippocampi D_mean_ (Gy)** | 8.04±0.21 | 7.93±0.16 | 7.99±0.19 | 8.03±0.20 | 7.97±0.14 |
|  | p=0.313 | p=0.383 | p=0.844 | p=0.641 |  |
| **Lens D_max_ (Gy)** | 4.99±0.16 | 4.79±0.24 | 4.09±0.59 | 3.25±0.83 | 2.82±1.10 |
|  | p=0.016 | p=0.016 | p=0.023 | p=0.25 |  |
| **Lens D_mean_ (Gy)** | 4.22±0.26 | 3.80±0.30 | 3.06±0.62 | 2.45±0.53 | 1.93±0.29 |
|  | p=0.008 | p=0.008 | p=0.008 | p=0.039 |  |
| **Eyes D_max_ (Gy)** | 25.40±3.09 | 26.05±3.40 | 24.86±3.37 | 22.37±5.28 | 23.56±5.17 |
|  | p=0.547 | p=0.383 | p=0.945 | p=0.383 |  |
| **Eyes D_mean_ (Gy)** | 7.34±0.56 | 7.12±1.00 | 6.23±0.93 | 5.37±1.07 | 4.96±0.94 |
|  | p=0.008 | p=0.016 | p=0.039 | p=0.641 |  |

significant at p＜0.05

**Supplementary Table 3** Summary of the p-values indicating the significance of differences in PTV and organs at risk between five groups

|  |  | **Group 1** | **Group 2** | **Group 3** | **Group 4** |
| --- | --- | --- | --- | --- | --- |
| **PTV D_mean_** | Group 2 | p=0.039 |  |  |  |
|  | Group 3 | p=0.016 | p=0.641 |  |  |
|  | Group 4 | p=0.055 | p=1 | p=0.641 |  |
|  | Group 5 | p=0.016 | p=0.055 | p=0.109 | p=0.055 |
| **PTV CI** | Group 2 | p=0.844 |  |  |  |
|  | Group 3 | p=0.742 | p=0.742 |  |  |
|  | Group 4 | p=0.547 | p=0.313 | p=0.844 |  |
|  | Group 5 | p=0.383 | p=0.742 | p=0.313 | p=0.109 |
| **PTV HI** | Group 2 | p=0.109 |  |  |  |
|  | Group 3 | p=0.042 | p=0.288 |  |  |
|  | Group 4 | p=0.23 | p=0.844 | p=0.383 |  |
|  | Group 5 | p=0.016 | p=0.041 | p=0.071 | p=0.039 |
| **PTV D_2cc_** | Group 2 | p=0.008 |  |  |  |
|  | Group 3 | p=0.063 | p=1 |  |  |
|  | Group 4 | p=0.109 | p=0.641 | p=0.641 |  |
|  | Group 5 | p=0.016 | p=0.25 | p=0.148 | p=0.641 |
| **PTV D_98%_** | Group 2 | p=0.109 |  |  |  |
|  | Group 3 | p=0.945 | p=0.383 |  |  |
|  | Group 4 | p=0.078 | p=0.148 | p=0.055 |  |
|  | Group 5 | p=0.742 | p=0.945 | p=0.843 | p=0.25 |
| **Cochea D_max_** | Group 2 | p=0.742 |  |  |  |
|  | Group 3 | p=0.055 | p=0.039 |  |  |
|  | Group 4 | p=0.461 | p=0.641 | p=0.382 |  |
|  | Group 5 | p=0.055 | p=0.109 | p=1 | p=0.313 |
| **Cochea D_mean_** | Group 2 | p=0.641 |  |  |  |
|  | Group 3 | p=0.055 | p=0.016 |  |  |
|  | Group 4 | p=0.25 | p=0.461 | p=0.547 |  |
|  | Group 5 | p=0.016 | p=0.109 | p=0.945 | p=0.25 |
| **OpticalNerve D_max_** | Group 2 | p=0.016 |  |  |  |
|  | Group 3 | p=0.008 | p=0.383 |  |  |
|  | Group 4 | p=0.008 | p=0.109 | p=0.641 |  |
|  | Group 5 | p=0.008 | p=0.039 | p=0.109 | p=0.313 |
| **OpticalNerve D_mean_** | Group 2 | p=0.023 |  |  |  |
|  | Group 3 | p=0.016 | p=0.945 |  |  |
|  | Group 4 | p=0.016 | p=1 | p=0.843 |  |
|  | Group 5 | p=0.547 | p=0.844 | p=1 | p=0.742 |
| **OpticChiasm D_max_** | Group 2 | p=0.023 |  |  |  |
|  | Group 3 | p=0.008 | p=0.547 |  |  |
|  | Group 4 | p=0.945 | p=0.023 | p=0.008 |  |
|  | Group 5 | p=0.109 | p=0.383 | p=0.461 | p=0.195 |
| **OpticChiasm D_mean_** | Group 2 | p=0.043 |  |  |  |
|  | Group 3 | p=0.148 | p=1 |  |  |
|  | Group 4 | p=1 | p=0.461 | p=0.547 |  |
|  | Group 5 | p=0.313 | p=0.195 | p=0.547 | p=0.148 |
| **Brainstem D_max_** | Group 2 | p=0.742 |  |  |  |
|  | Group 3 | p=0.461 | p=0.383 |  |  |
|  | Group 4 | p=0.844 | p=0.547 | p=0.641 |  |
|  | Group 5 | p=0.742 | p=0.742 | p=0.312 | p=0.945 |
| **Brainstem D_mean_** | Group 2 | p=0.008 |  |  |  |
|  | Group 3 | p=0.641 | p=0.742 |  |  |
|  | Group 4 | p=0.945 | p=0.25 | p=0.641 |  |
|  | Group 5 | p=0.844 | p=0.195 | p=0.461 | p=0.742 |
| **Pituitary D_max_** | Group 2 | p=0.039 |  |  |  |
|  | Group 3 | p=0.078 | p=0.313 |  |  |
|  | Group 4 | p=0.109 | p=0.742 | p=0.945 |  |
|  | Group 5 | p=0.039 | p=0.945 | p=0.844 | p=0.742 |
| **Pituitary D_mean_** | Group 2 | p=0.945 |  |  |  |
|  | Group 3 | p=0.612 | p=0.461 |  |  |
|  | Group 4 | p=0.844 | p=1 | p=0.148 |  |
|  | Group 5 | p=0.844 | p=0.25 | p=0.945 | p=0.641 |
| **Hippocampi D_max_** | Group 2 | p=0.383 |  |  |  |
|  | Group 3 | p=0.039 | p=0.195 |  |  |
|  | Group 4 | p=0.055 | p=0.383 | p=0.641 |  |
|  | Group 5 | p=0.008 | p=0.016 | p=0.148 | p=0.039 |
| **Hippocampi D_mean_** | Group 2 | p=0.25 |  |  |  |
|  | Group 3 | p=0.461 | p=0.461 |  |  |
|  | Group 4 | p=0.945 | p=0.383 | p=0.945 |  |
|  | Group 5 | p=0.313 | p=0.383 | p=0.844 | p=0.641 |
| **Lens D_max_** | Group 2 | p=0.109 |  |  |  |
|  | Group 3 | p=0.008 | p=0.008 |  |  |
|  | Group 4 | p=0.008 | p=0.008 | p=0.023 |  |
|  | Group 5 | p=0.016 | p=0.016 | p=0.023 | p=0.25 |
| **Lens D_mean_** | Group 2 | p=0.109 |  |  |  |
|  | Group 3 | p=0.008 | p=0.008 |  |  |
|  | Group 4 | p=0.008 | p=0.008 | p=0.016 |  |
|  | Group 5 | p=0.008 | p=0.008 | p=0.008 | p=0.039 |
| **Eyes D_max_** | Group 2 | p=0.742 |  |  |  |
|  | Group 3 | p=0.844 | p=0.945 |  |  |
|  | Group 4 | p=0.313 | p=0.195 | p=0.461 |  |
|  | Group 5 | p=0.547 | p=0.383 | p=0.945 | p=0.383 |
| **Eyes D_mean_** | Group 2 | p=0.461 |  |  |  |
|  | Group 3 | p=0.008 | p=0.055 |  |  |
|  | Group 4 | p=0.016 | p=0.016 | p=0.039 |  |
|  | Group 5 | p=0.008 | p=0.016 | p=0.039 | p=0.641 |

**Supplementary Table 4** Overview of PTV length, hippocampi length, and hippocampi angle for each patient.

|  |  | **hippocampi angle** | **PTV length** | **hippocampi length** |
| --- | --- | --- | --- | --- |
| **Patient 1** | Group 1 | 48.577 | 15 | 2.75 |
|  | Group 2 | 45.678 | 14.25 | 3 |
|  | Group 3 | 41.104 | 13.5 | 3 |
|  | Group 4 | 38.504 | 13 | 3.75 |
|  | Group 5 | 39.338 | 13.5 | 3.5 |
| **Patient 2** | Group 1 | 48.631 | 14.75 | 2.5 |
|  | Group 2 | 43.975 | 14.25 | 2.75 |
|  | Group 3 | 47.024 | 13.75 | 3.5 |
|  | Group 4 | 45.955 | 13.25 | 4 |
|  | Group 5 | 43.130 | 15 | 3.75 |
| **Patient 3** | Group 1 | 48.198 | 14.25 | 2.75 |
|  | Group 2 | 47.114 | 13.75 | 3 |
|  | Group 3 | 47.208 | 13.25 | 2.25 |
|  | Group 4 | 43.605 | 12.75 | 3.5 |
|  | Group 5 | 44.284 | 13.75 | 3 |
| **Patient 4** | Group 1 | 48.964 | 15.5 | 2.25 |
|  | Group 2 | 47.758 | 14.25 | 2.75 |
|  | Group 3 | 45.048 | 13.5 | 2.25 |
|  | Group 4 | 43.610 | 13.5 | 3.5 |
|  | Group 5 | 48.259 | 14 | 2.25 |
| **Patient 5** | Group 1 | 47.312 | 14.75 | 3.25 |
|  | Group 2 | 40.504 | 14.25 | 3.25 |
|  | Group 3 | 35.517 | 13.25 | 3.25 |
|  | Group 4 | 36.267 | 13.25 | 4 |
|  | Group 5 | 33.963 | 13.75 | 3.75 |
| **Patient 6** | Group 1 | 47.302 | 15.5 | 2.75 |
|  | Group 2 | 47.856 | 15 | 2.75 |
|  | Group 3 | 41.703 | 13.75 | 3.5 |
|  | Group 4 | 56.326 | 14 | 2.75 |
|  | Group 5 | 54.562 | 13.5 | 3.25 |
| **Patient 7** | Group 1 | 45.345 | 14.5 | 2.5 |
|  | Group 2 | 47.383 | 13.5 | 2.75 |
|  | Group 3 | 46.327 | 12.75 | 3.25 |
|  | Group 4 | 43.029 | 13.25 | 3.25 |
|  | Group 5 | 43.685 | 14 | 3.5 |
| **Patient 8** | Group 1 | 47.153 | 14.5 | 3.5 |
|  | Group 2 | 45.588 | 14 | 3.25 |
|  | Group 3 | 43.390 | 13.5 | 3.75 |
|  | Group 4 | 42.592 | 13.75 | 4.25 |
|  | Group 5 | 40.703 | 14 | 4 |

**Supplementary Table 5** The correlation assessment of two linear regression models

|  | **R** | **Durbin–Watson** | **ANOVA F** | **ANOVA significance** |
| --- | --- | --- | --- | --- |
| **Model 1** | 0.486 | 1.887 | 11.748 | 0.001 |
| **Model 2** | 0.335 | 1.820 | 4.796 | 0.035 |

Model 1 is the model for the maximum dose to the hippocampi and PTV length.

Model 2 is the model for the mean dose to the hippocampi and hippocampi length.
